# sRNA OsiA Stabilizes Catalase mRNA during Oxidative Stress Response of *Deincoccus radiodurans* R1

**DOI:** 10.3390/microorganisms7100422

**Published:** 2019-10-08

**Authors:** Yun Chen, Dong Xue, Wenjie Sun, Jiahui Han, Jiang Li, Ruyu Gao, Zhengfu Zhou, Wei Zhang, Ming Chen, Min Lin, Jin Wang, Kaijing Zuo

**Affiliations:** 1 Department of Plant Science, School of Agriculture and Biology, Shanghai Jiao Tong University, Shanghai 200240, China; chenyun0402ye@163.com (Y.C.); sunwenjie_007@163.com (W.S.); 2 Biotechnology Research Institute, Chinese Academy of Agricultural Sciences, Beijing 100081, China; xue_dong_kevin@126.com (D.X.); 13121257599@163.com (J.H.); rainie0327@mail.dlut.edu.cn (R.G.); zhouzhengfu@caas.cn (Z.Z.); zhangwei01@caas.cn (W.Z.); chenming01@caas.cn (M.C.); linmin57@vip.163.com (M.L.)

**Keywords:** *Deinococcus radiodurans R1*, small nonconding RNAs, oxidative stress, OsiA

## Abstract

*Deinococcus radiodurans* adapts to challenging environments by modulating gene expression in response to oxidative stress. Recently, bacterial small noncoding RNAs (sRNAs) have been presumed to participate in the transcriptional or translational regulation of stress-responsive genes. We found 24 sRNAs that may be involved in the oxidative stress response of *D. radiodurans* by deep RNA sequencing. Moreover, a typical stress-inducible sRNA, IGR_3053, named OsiA, was predicted to bind to the mRNA of *katA*, *katE*, and *sodC* by the bioinformatics method. An *osiA* knockout of *D. radiodurans* displayed increased sensitivity to H_2_O_2_ and the decreased catalase activity and total antioxidant activity, suggesting that OsiA probably serves as a regulator in the adaptation to oxidative environments. Further microscale thermophoresis results demonstrated that OsiA can directly bind to the mRNA of *katA*, *sodC*, and *katE*. The stability test result of *katA* mRNA showed that its half-life was 2 min in the *osiA* mutant compared with 5 min in the wildtype(wt) strain. Our results indicated that OsiA can enhance the stability of *katA* mRNA and the activity of KatA and consequently the oxidation resistance of *D.radiodurans*. We are the first one to explore the super-strong oxidative stress resistance of *D.radiodurans* at the level of post-transcriptional regulation, and found a new pathway that provides a new explanation for the long-term adaptability of *D.radiodurans* in extreme environments.

## 1. Introduction

*Deinococcus* bacteria are extremophiles known for their superior resistance to ionizing radiation and oxidative stresses [1,2,3,4,5]. Decades of studies have indicated that their extreme resistance is attributed to the protection of oxidized protein degradation and activation of multiple factors and mechanisms for repairing DNA damage [6,7,8,9]. In the DNA repair system of *Deinococcus*, RecA(Recombinase A), RecFOR system, DNA glucoamylase, and other *Deinococcus*-specific proteins such as DdrB(DNA damage response protein) and PprA(DNA damage repair protein) show higher repair activity than *Escherichia coli* in maintaining genomic stability [10]. When facing oxidative stresses, *Deinococcus* bacteria have developed a more efficient ROS(Reactive Oxygen Species)-scavenging system to defend against oxidative stress in comparison with other stress-sensitive bacteria [5]. This well-equipped system includes catalases (KatA, KatE, SodC, etc.), antioxidant defense proteins (MsrA, MsrB, etc.), specific metabolites such as carotene and carotenoids [9,10,11,12,13], and a significantly higher Mn/Fe concentration ratio. In addition, some stress response transcriptional factors like CarD(RNA polymerase-interacting regulator), DrRRA(DNA-binding response regulator A), IrrE(Ionizing radiation resistance protein), OxyR have also been reported to play significant roles in the stress resistance of *D. radiodurans*. Aside from these factors, *Deinococcus* bacteria have developed a conserved regulon, DdrO and PprI (also named as IrrE), that is involved in transcriptional regulation during the oxidative response [14,15]. DdrO and PprI serve as a global regulator pair that regulates a vast array of genes involved in diverse cellular processes under stress conditions, indicating the complexity of the stress tolerance mechanism in *Deinococcus* [10]. This has prompted us to investigate whether there are also other factors that cooperatively regulate the expression of dozens of genes in *Deinococcus radiodurans*.

Oxidative stress reaction becomes the secondary of all stress responses on account of excessive ROS [16]. Thus, the key to improving the biological resistance to adverse environments such as high temperature, high salt, drought, and radiation is to remove the excessive ROS. Catalases are major players among the ROS scavenging pathway [16]. The three members, Kat, Sod, and Pod, work in different divisions; regarding Kat, it is capable of reducing peroxide radicals to molecular oxygen and water; superoxide radicals are reduced to peroxide by Sod; and the role of Pod is peroxide detoxification [17]. So far, substantive investigations have been performed with regard to their post-transcriptional regulation and there is still a lot of space left for their regulation among diverse metabolic pathways. A noncoding RNA RyhB has previously been reported to degrade mRNAs encoding iron binding proteins like the mRNA of *sodB*, which encodes Fe-superoxide dismutase [18]. Nfis, a previously reported sRNA involved in the nitrogen fixation of *Pseudomonas stutzeri* A1501 were proven to regulate oxidative stress response by binding to the mRNA of the catalase coding gene *katB* [19,20]. However, there have been no reports on the post-transcriptional regulation of catalases in *D.radiodurans*. Current studies have focused on DNA and protein levels, few reports have been reported on the regulation of the post-transcriptional level. There is still a lack of adequate explanation for the super extreme environmental adaptability of *D.radiodurans*.

Small noncoding RNAs (sRNAs) in bacteria are diverse in sequence, and are perceived as crucial elements in gene regulation [21]. sRNAs usually have a length of 50–500 nt, and most regulate mRNA stability or translation efficiency through base-pairing [22]. sRNAs may be classified into three categories according to the location of gene targeting: *trans*-encoded, *cis-*internal, and intergenic sRNAs (in a noncoding region between two genes) [23]. The majority of bacterial sRNAs are *trans*-encoded sRNAs that serve as important regulatory factors [24]. Regulatory sRNAs are frequently induced in stress responses, and they bind to mRNA of the aforementioned genes encoding the transcription factors and proteins to some extent [25], which together contribute to the ability of cells to cope with specific growth conditions such as irradiative or drought conditions. Few studies have investigated the sRNA regulation of *D.radiodurans*. A total of 144 noncoding RNAs were detected during gamma-irradiated phases of *D.radiodurans* and 199 noncoding transcripts were detected in the *D.radiodurans* genome under normal condition; nevertheless, their role in stress resistance has not been studied in depth [26].

A previous study determined the transcriptome of *D. radiodurans* under 20 mM H_2_O_2_ treated for 30 min [13]; interestingly, we found that a relatively obvious phenotype appeared after 80 mM H_2_O_2_ treated for 30 min, therefore we determined the transcriptome of *D. radiodurans* under such conditions. Combined with the transcriptome data with bioinformatics analysis, one sRNA attracted our attention for its binding to the mRNAs of the KatA, KatE, and SodC coding genes. It is speculated that it may be involved in several metabolic pathways of the oxidative stress response of *D. radiodurans*, so the biological function was studied in detail.

## 2. Materials and Methods

### 2.1. Bacteria Strain, Growth Conditions, and Oxidative Stress Treatment

*D. radiodurans* cells were purchased from China General Microbiological Culture Collection Center(CGMCC, Beijing, China) and were cultured in TGY medium (1% tryptone, 0.5% yeast extract, and 0.1% glucose, pH 5.3, OXIOD, Hampshire, United Kingdom) at 30 °C and shaken at 220 rpm. When the OD (optical density) of the samples reached 0.8 (7 × 10^6^ CFU/mL approximately), the culture was treated with 80 mM H_2_O_2_. After 30 min, the bacteria cells were centrifuged at 7000 rpm for 10 min, and the pellets were washed twice with PBS buffer (Phosphate Buffer Saline, 0.02% KH_2_PO_4_, 0.29% Na_2_HPO_4_·12H_2_O, 0.8% NaCl, 0.02% KCl, pH 7.5) and finally centrifuged at 12,000× *g* for 1 min. An untreated cell culture grown under the same condition was used as the control.

### 2.2. Total RNA Extraction

Total RNA was extracted according to the protocol of the PureLink^TM^ RNA Mini Kit (Invitrogen, California, USA) and purified decontaminated using a genomic DNA eraser (Tigan, Beijing, China). The RNA concentration was measured using a Qubit RNA Assay Kit in a Qubit 3.0 Fluorometer (Life Technologies, California, USA), and RNA quality and concentration were assessed using the RNA Nano 6000 Assay Kit of the Bioanalyzer 2100 system (Agilent Technologies, California, USA).The RNA sample was diluted to 1000 ng/µL for RNA-Seq.

### 2.3. Candidate sRNA Screen and the Prediction of sRNA Target Genes

The raw data from the Illumina sequencing platform (https://www.illumina.com/ Illumina/sequencing and array-based solutions for genetic research, David Walt and Larry Bock, April, 1998) [27] was preprocessed to remove the adapter sequences and filter out the low-quality data for QC purposes. The reference *D. radiodurans* R1 genome and gene annotations were downloaded directly from the NCBI FTP website [28]. The sRNA screen was performed according to the method described by Zhouwei Chen et al. When the ratio between the MEV (mean expression value) of transcripts in the intergenetic region and their adjacent CDS (Coding sequence) was greater than 2, the transcripts were considered to be an sRNA candidate [29]. The sRNA was further validated by PCR. The RNA interaction prediction tool IntaRNA (http://rna.informatik.uni-freiburg.de/IntaRNA/Input.jsp?jobID=4476547&reload=true IntaRNA-RNA-RNA interaction, Busch, 2008) [30] was used to predict sRNA targets online.

### 2.4. Gene Ontology (GO) and KEGG Enrichment Analysis

Gene ontology (GO) annotations (http://geneontology.org/ The Gene Ontology Resource, Michael Ashburner, May, 2000) [31] were conducted using the AgBaseGOanna package60 (http://agbase.msstate.edu/GOAnna.html Fiona M. McCarthy, 2006) with a BLASTx (https://blast.ncbi.nlm.nih.gov/Blast.cgi?PROGRAM=blastx&PAGE_TYPE=BlastSearch&LINK_LOC=blasthome Basic Local Alignment Search Tool, Jun, 2005) [32] search against the *D. radiodurans* R1 genome database. GO enrichment of differentially expressed genes was analyzed by using a Perl module (GO::TermFinder) (http://go.princeton.edu/cgi-bin/GOTermFinder Generic Gene Ontology Term Finder, Gavin Sherlock, Nov, 2009) [33]; KOBAS online database (KEGG Orthology Based Annotation System, http://kobas.cbi.pku.edu.cn/ Jianmin, July, 2006) [34]. It was concluded that GO items with corrected *p* values less than 0.05 were significantly enriched. The KOBAS online database was applied to enrich the DEGs. The KEGG (Kyoto Encyclopedia of Genes and Genomes, https://www.kegg.jp/ KEGG: Kyoto Encyclopedia of Genes and Genomes, Kanehisa Laboratories, 1995) [35] pathways with a modified *p* value less than 0.05 were considered to be significantly enriched in the tested pathways.

### 2.5. Microscale Thermophoresis Analysis

The microscale thermophoresis (MST) technique was used to quantify the interactions between sRNAs and the mRNAs transcribed by the target genes [36,37,38]. A T7 transcription kit was first used to synthesize the 5′FAM-labeled mRNA of the target genes (*katA*, *katE*, *sodC*, etc.) in vitro [34]. The labeled mRNAs were then diluted to the fluorescence range of 200–1000 according to the protocol in the user manual of the Monolith NT.115 (Nano temper company, MÜnchen, Germany).

sRNA (50 µg) was used as a template for in vitro transcription, and the resulting synthetic mRNA was dissolved in DPEC(diethylpyrocarbonate)-treated water to an OD = 2. sRNA (10 µL) was first added to a PCR tube, and then serially diluted with DPEC-treated water using a 1:1 ratio for the next 14 tubes. After that, 10 µL of labeled mRNA was mixed with the diluted sRNAs in each tube. After incubation for 30 s, the samples were loaded into MST NT.115 standard glass capillaries. The molecular interactions were tested and the Kd (dissociation equilibrium constant) values were calculated using the NanoTemper software (Nano Temper company, MÜnchen, Germany) package.

## 3. Results

### 3.1. Deep Sequencing sRNAs from D. radiodurans R1 under Oxidative Stress

To globally identify small noncoding RNAs differentially expressed in response to oxidative stress in *D. radiodurans*, we exposed at least three replicate cultures of *D. radiodurans* to 80 mM H_2_O_2_. Total RNA was isolated from untreated cultures (CDR) and H_2_O_2_-treated cultures (TDR). sRNAs were sequenced using a strand-specific size-selected (50–500 nt) sRNA library preparation.

After trimming the adapters and discarding the low-quality reads, there were about 6 Gbp reads for each sample to evaluate the gene expression and predict sRNAs. The valid data of CDR and TDR were 79.77% and 77.92%, respectively (Supplementary Table S1). The transcriptional reads were mapped to the *D. radiodurans* genome to identify sRNAs that are antisense to the structure genes. As shown in Supplementary Figure S1, sRNAs in *D. radiodurans* genome ranged from 50 to 500 bp, and most of the sRNAs were from 50 to 200 bp. As the length increased, the number of sRNAs was greatly reduced, indicating that sRNAs regulate the expression of target genes mainly via binding with short sequences less than 200 bp.

To confirm the transcriptomic data, we randomly selected 10 sRNAs to validate their expression levels under the oxidative condition by qRT-PCR (quantitative real-time PCR). As shown in Supplementary Figure S1, the expression of 10 sRNAs quickly responded to oxidative treatment and were in accordance with the RNA-Seq analysis.

To make a thorough inquiry of sRNA function in gene regulation, we analyzed the whole genome using computer prediction and experimental methods to identify transcriptional regulatory elements.

Further functional classification of target genes showed that the target genes of 97 sRNAs act as enzymes (42 upregulate and 55 downregulate), the target genes of 32 sRNAs act as kinases (15 upregulate and 17 downregulate), the target genes of 21 sRNAs act as transcription factors (seven upregulate and 14 downregulate), and the target genes of 22 sRNAs are structural proteins (nine upregulate and 13 downregulate) (Table 1). This result indicates that sRNAs can regulate the expression of both structural genes and regulators (kinases and transcription factors) at the transcriptional level. What we found very amusing about this result is that one sRNA may regulate more than one target gene with diverse functions.

### 3.2. 24 Oxidative Related sRNAs Were Identified by KEGG Analysis

To analyze the function of target genes during oxidative responses, we used the KOBAS database to enrich the KEGG pathway that is probably regulated by sRNAs. The enriched results showed that the target genes regulated by 24 sRNAs were involved in six oxidative stress response relevant metabolism pathways (*p* < 0.05) including peroxisome function, DNA mismatch repair, glutathione metabolism, carotenoid biosynthesis, the two-component system, and vitamin B6 metabolism (Table 2, Supplementary Tables S2 and S3). It also showed that the enriched pathways were regulated by multiple sRNA regulators. For example, seven sRNAs are involved in the peroxisome pathway, 15 sRNAs are involved in the DNA mismatch repair pathway, and eight sRNAs are involved in glutathione metabolism. Hence, these results indicate that oxidative stress-inducible sRNAs have regulatory functions in the expression of the genes related to robust repair systems and specialized metabolism in *D. radiodurans*, and the cooperation of different repair systems or stress signaling pathways may be tuned through the same sRNA.

It was noted that one sRNA, OsiA (Oxidative stress-induced sRNA A), which is located in the 358,768 to 358,969 region of chromosome 2, can target five genes related to six pathways including peroxisome, mismatch repair, and two-component system (Table 2). In the peroxisome functional pathway, three genes coding KatA (DR_1998), SodC (DR_A0202), and KatE (DR_A0259) were the target genes regulated by OsiA. Furthermore, the gene coding RplV(DR_0316) in mismatch repair and the gene coding DR_A0354 are probably also regulated by OsiA. These results indicate that OsiA may play a crucial role in transcriptional regulation in *D. radiodurans* upon oxidative stress.

### 3.3. Characterization of OsiA, an Oxidative Response sRNA

**Figure 1:**
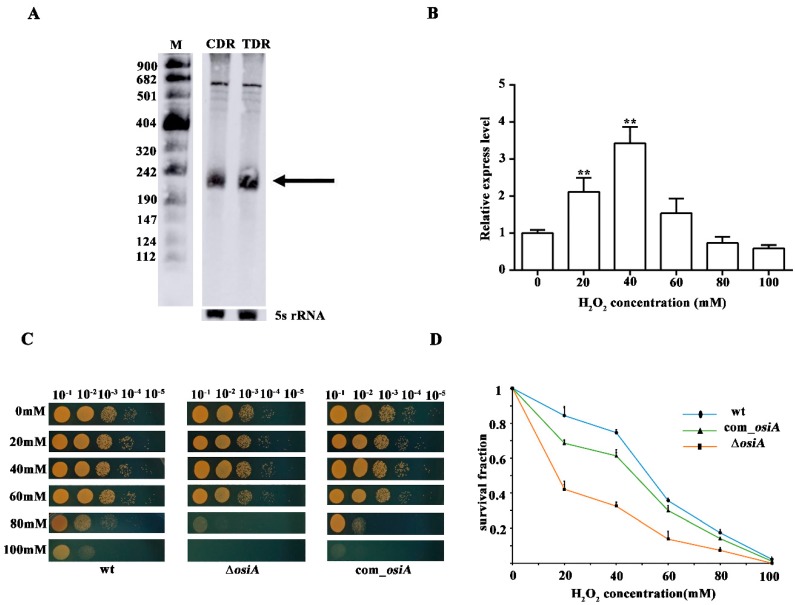
Stress tolerance analysis of OsiA. (**A**) Northern blotting identification of OsiA. CDR is the control conditions, while TDR is the H_2_O_2_-treated conditions, with the arrow indicating OsiA, while 5S rRNA bands are shown as loading controls. (**B**) The expression profile of OsiA. ** means that the expression of OsiA is significantly different from that of the wild-type strain, that is, the *p* value is less than 0.01. (**C**) The phenotypic analysis of *osiA* knockout mutant, the *osiA* complementary strain, and the control under different H_2_O_2_-treated conditions. (**D**) Survival growth analysis of the *osiA* knockout mutant, *osiA* complementary strain, and the control under different H_2_O_2_-treated conditions.

Northern blotting showed that the expression of OsiA was increased in TDR compared with CDR, which further verified the reliability of the transcriptome data (Figure 1A). qRT-PCR showed that OsiA expression was first increased and later decreased with an increase in H_2_O_2_ concentration, and the maximum expression was reached at 40 mM, which suggested that OsiA responded to hydrogen peroxide shock (Figure 1B). To clarify the function of OsiA in the oxidative stress response, we knocked out *osiA* in *D. raidodurans* and analyzed the oxidative phenotype of this mutant. Under normal conditions, there was no difference between the WT, ∆*osiA*, and *osiA* complementary strain. With the increase of H_2_O_2_ concentration, ∆*osiA* was more sensitive than WT (Figure 1C), and the survival curve demonstrated that ∆*osiA* could not survive with a high concentration of H_2_O_2_ (Figure 1D), while the phenotype and survival fraction of *osiA* mutant could be recovered after supplementing *osiA* in the mutant strain, which indicates that OsiA is necessary for the growth of *D. radiodurans* under oxidative conditions.

**Figure 2:**
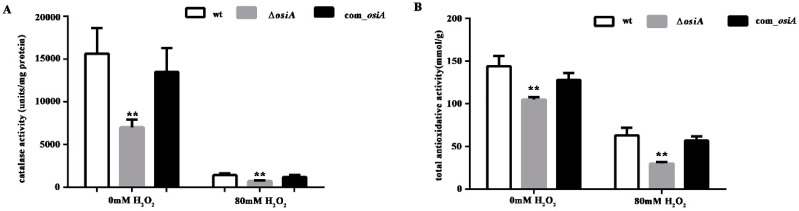
Catalase activity and total antioxidative activity of the wild type strain and *osiA* mutant strain. (**A**) Catalase activity of the wt, ∆*osiA*, and *osiA* complementary strain. (**B**) Total antioxidative activity of the wt, ∆*osiA*, and *osiA* complementary strain. ** means the difference is very significant compared with the control group, that is, the *p* value is less than 0.01.

To further quantify the oxidative resistance of *D. radiodurans* in the absence of *osiA*, catalase activity (Figure 2A) and total antioxidant capacity (Figure 2B) in wild type, *osiA* mutant, and *osiA* complementary strains were measured. The results revealed that both catalase activity and total antioxidant capacity were significantly reduced and the compensatory strain can compensate for this result to some extent.

### 3.4. OsiA Enhances the Stability of Transcripts by Binding to the Target Genes through Multiple Bases

Interestingly, the bioinformatics analysis showed that the binding sequences of OsiA and the transcripts of the three target genes were all located in the same stem-loop (nucleotides 162–175) of OsiA (Supplementary Figure S3).

**Figure 3:**
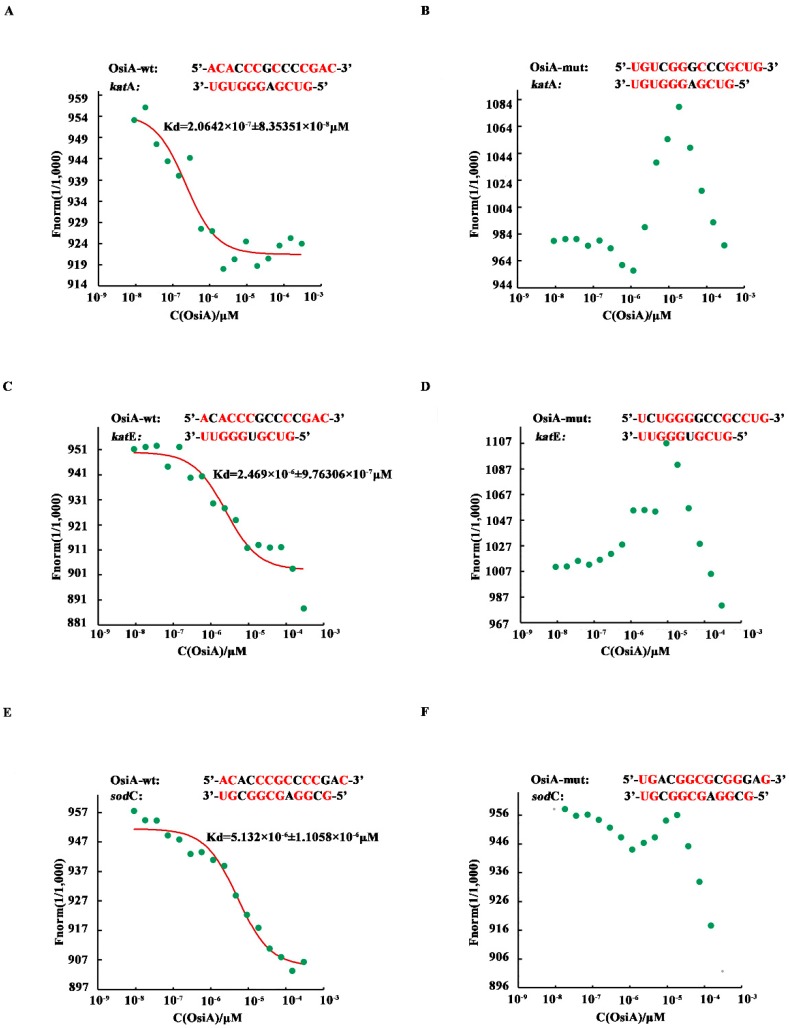
Microscale thermophoresis of selected sRNAs and their target genes. Red bases mean complementary bases in the binding sequence, the red curve is the fitted combination curve, and the Kd (dissociation equilibrium constant) value is the binding constant of sRNAs and their targets. (**A**,**C**,**E**) are the binding of *dr_1998*(*katA*) mRNA, *dr_A0202*(*sodC*) mRNA, and *dr_A0259*(*katE*) mRNA, respectively, with OsiA-wt. (**B**,**D**,**F**) are the binding of *dr_1998* mRNA, *dr_A0202* mRNA, *dr_A0259* mRNA, respectively, with OsiA-mut.

To investigate whether OsiA binds directly to the target genes, microscale thermophoresis was applied to identify the binding strength between OsiA or its mutants and the corresponding complementary fragment in the mRNA of target genes (*katA*, *katE*, *sodC*). Among the tested genes, *katA* mRNA had the strongest binding force with OsiA. The interaction of *katE* mRNA and OsiA was ranked second, while *sodC* mRNA had the weakest binding with OsiA. According to the predicted binding strength in different combinations, we mutated different bases in OsiA and then assayed the change in binding strength (Figure 3A,C,E). As expected, the binding strength between the mutated OsiA variants and their target genes was completely disrupted (Figure 3B,D,F), suggesting that OsiA strongly binds to the target genes through different bases. Using *katA* mRNA as an example, when the binding sequence ACACCCGCCCCGAC of OsiA was replaced by the mutated sequence UGUCGGGCCCGCUG, no binding could be detected between OsiA and *katA* mRNA.

The binding assay showed that OsiA can regulate multiple genes in different cellular antioxidant pathways, indicating that OsiA is required for adaptation to extreme environments and probably has specific regulatory functions in *D. radiodurans*.

**Figure 4:**
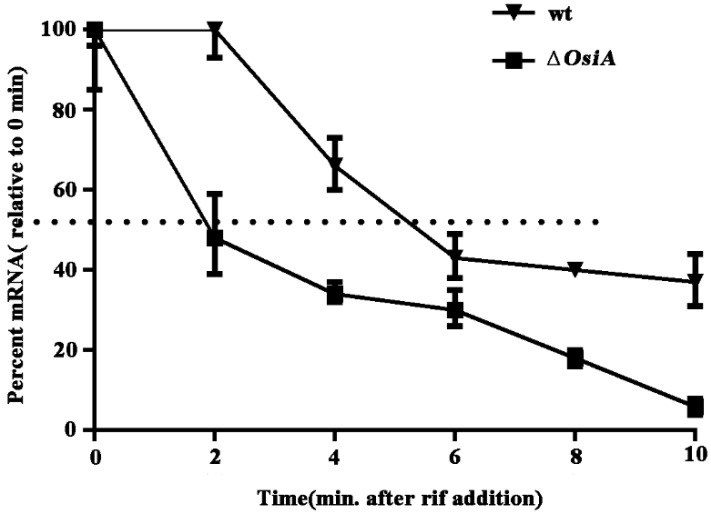
Halflife of *katA* mRNA in the wildtype strain and *osiA* mutant strain.

The half-life of *katA* mRNA in the wildtype strain was 5 min, but this decreased to 2 min with *osiA* knocked out under the H_2_O_2_ treated condition (Figure 4). This result indicates that OsiA regulated *katA* mRNA at the posttranscriptional level and enhanced the stability of *katA* mRNA.

## 4. Discussion

*D. radiodurans* is a desiccation- and radiation-tolerant bacterium [2,3,4,5]. In recent years, many studies have examined the molecular mechanism of *D.radiodurans* in adapting to extreme environments [6,7,8,9]. High-resolution RNA-Seq analyses have indicated that the bacterial regulatory factors include transcription factors, protein kinases, and noncoding RNAs. When facing unfavorable environmental conditions, *D. radiodurans R1* effectively activates the DNA repair system to protect DNA from breaking, or enhances the expression of antioxidants to protect proteins from degradation [1,10]. In particular, sRNA-mediated transcriptional regulation is likely to finely regulate stress responses to environmental challenges such as oxidative stress.

Peroxisome is a multifunctional organelle involved in the generation and decomposition of ROS, and carotenoid deinoxanthin is an important chemical involved in resistance against extreme oxidative stresses in *D. radiodurans* [10,11]. Furthermore, a growing number of studies show that two-component systems play an irreplaceable role in the sensing of environmental changes and adapting to adverse environments [39,40]. Vitamin, a kind of small molecular substance, can assist peroxidase to resist UV damage, reduce ROS content, and enhance oxidative stress resistance [41,42]. We identified 24 sRNAs involved in these six metabolic pathways and we will lucubrate them to fully understand how they participate in the oxidative stress and even defense responses of *D.radiodurans*, and finally construct the post-transcriptional regulatory network of *D.radiodurans*.

As previously reported, there are many transcriptional regulators involved in the abiotic stress of *D.radiodurans* such as DrRRA, OxyR, and IrrE. DrRRA was reported to regulate KatA and SodC, and both DrRRA and OxyR have been reported to regulate KatE in oxidative stress response. As shown in our results, OsiA interacts with the mRNA of *katA*, *katE*, and *sodC*, so we can infer that OsiA may have similar functions in global regulation.

The upstream gene (*dr_A0243*) of *osiA* encodes a flavohemoprotein that utilizes O_2_ and NAD(P)H to convert NO into nitrates [43]. The downstream gene (*dr_A0244*) of *osiA* encodes a protein that is very similar to the radical S-adenosyl-L-methionine protein, and acts against a variety of toxic agents caused by oxidative stress [44,45]. The remediating functions of the two genes, along with the target gene analysis of OsiA, suggest that OsiA is a pivotal sRNA during the oxidative response in *D. radiodurans*.

The interaction results showed that all target genes shared complementary sequences with OsiA at 162–175 nt (Supplementary Figure S2B), while it can be seen from the binding analysis that OsiA targets genes by different recognition sites (Figure 3). Future studies are needed to mutate the different sites in OsiA and explore its other targets to elucidate the detailed function of OsiA and how it crosstalks with other regulators during the adaptation of extremophiles to extreme environments.

A series of reports have focused on the extreme resistance mechanism of *D.radiodurans*. So far, its extreme resistance can be attributed to the following: its own genome structure, antioxidant enzymes, stress protective protein, carotene biosynthesis, high Mn/Fe ratio, and transcriptional regulator. No one has reported on the post-transcriptional regulation of these resistance genes. This study was conducted to explore the post-transcriptional regulation of *D.radiodurans* and reveal its long term extreme environmental adaptability from a new perspective. In the future, we will further study the post-transcriptional regulation of *D.radiodurans*, adding a strong note to the blueprint of its complex extreme environmental resistance mechanism.

## Figures and Tables

**Table 1:** Molecular function of sRNA target genes.

Function	sRNAs (+)	sRNAs (−)
Kinases	15	17
Transcription factor	7	14
Enzymes	42	55
Structural protein	9	13

**Table 2:** Functional annotation of oxidative response sRNAs in the KEGG pathway.

sRNA Name	Pathway Mane	Target Genes Products
IGR_3053	Peroxisome	KatA, SodC
	Mismatch repair	RplV
	Two component system	methyl-accepting chemotaxis protein
IGR_2205	Peroxisome	class V aminotransferase
	Mismatch repair	DNA ligase, DNA polymerase III, tau/gamma subunit, DR_2586
	Glutathione metabolism	glucose-6-phosphate 1-dehydrogenase
	Carotenoid metabolism	phytoene dehydrogenase
	Two component system	NatA, DR_A0009, methyl-accepting chemotaxis-like protein
	Vitamin B6 metabolism	pyridoxamine 5-phosphate oxidase
IGR_1662	Peroxisome	KatE,
	Mismatch repair	MutT, Ssb, MutS
	Carotenoid metabolism	phytoene synthase
	Two component system	5-(carboxyamino)imidazole ribonucleotide mutase
IGR-1449	Mismatch repair	MutL, DNA helicase II
	Glutathione metabolism	potassium-transporting ATPase subunit C
	Two component system	potassium-transporting ATPase subunit C, KdpD-related protein
	Vitamin B6 metabolism	RpsO, PdxT
IGR_1916	Peroxisome	long-chain fatty acid--CoA ligase
	Mismatch repair	DR_1244
	Glutathione metabolism	ValS
IGR_2408	Mismatch repair	3-oxoacyl-acyl carrier protein reductase
	Two component system	methyl-accepting chemotaxis protein, CheA-related protein
	Vitamin B6 metabolism	threonine synthase
IGR_1612	Mismatch repair	DNA polymerase III subunit epsilon, RuvA
IGR_2590	Peroxisome	acyl-CoA synthetase
	Mismatch repair	3-isopropylmalate dehydratase large subunit
	Vitamin B6 metabolism	pyridoxamine kinase
IGR_884	Mismatch repair	XseA
	Glutathione metabolism	diaminopimelate decarboxylase
	Two component system	potassium-transporting ATPase subunit B
IGR_771	Glutathione metabolism	nitrogen regulatory protein P-II, leucyl aminopeptidase
	Carotenoid biosynthesis	lycopene cyclase
IGR_2060	Two component system	potassium-transporting ATPase subunit A
	Vitamin B6 metabolism	RuvB, PdxS
IGR_1174	Peroxisome	isocitrate dehydrogenase
	Glutathione metabolism	isocitrate dehydrogenase
IGR_2150	Mismatch repair	short chain dehydrogenase/reductase family oxidoreductase
	Glutathione metabolism	cephalosporin acylase
IGR_2479	Mismatch repair	DNA polymerase III subunit alpha
	Two-component system	succinate dehydrogenase, cytochrome subunit
IGR_2389	Peroxisome	Cu/Zn family superoxide dismutase
IGR_76	Mismatch repair	single-stranded DNA-binding protein
IGR_585	Mismatch repair	RpsS
IGR_2012	Mismatch repair	single-stranded-DNA-specific exonuclease
IGR_2690	Glutathione metabolism	Arginase, 6-phosphogluconate dehydrogenase-like protein
IGR_373	Two-component system	NADH dehydrogenase II
IGR_826	Two-component system	SdhB
IGR_1951	Two-component system	DNA-binding response regulator
IGR_388	Two-component system	PilH

## Supplementary Materials

Supplementary Materials can be found at https://www.mdpi.com/2076-2607/7/10/422/s1. Table S1: Small RNA sequencing and classification in *D. radiodurans* R1, Table S2: Information of 24 sRNAs involved in oxidative stress response, Table S3: KEGG pathway analysis of oxidative response sRNAs in *D. radiodurans R1,* Figure S1: The sRNA length distribution in *D. radiodurans R1*. The sRNA length (nt) is shown on the x-axis and the percentage of sRNA quantity is represented on the y-axis, Figure S2: qRT-PCR validation of 10 sRNAs identified by RNA-seq. Vertical values represent fold change (log_2_) of small noncoding RNAs (sRNAs) at the transcriptional level after H_2_O_2_ treatment, Figure S3: The deduced second structure of OsiA. The black line in the figure represents the common areas on OsiA where each target gene is combined. The color legends represent base pair probabilities.
