# Supplementary material for: sRNA OsiA Stabilizes Catalase mRNA during Oxidative Stress Response of Deincoccus radiodurans R1
Source: Microorganisms. 2019 Oct 8;7(10):422. doi: 10.3390/microorganisms7100422 (PMC6843392; doi:10.3390/microorganisms7100422)
Supplement: Supplementary file 1 [file microorganisms-07-00422-s001.pdf]

sRNA OsiA stabilizes catalase mRNA during oxidative stress response of *Deincoccus radiodurans* R1

Yun Chen[1,2], Dong Xue[2], Wenjie Sun[1], Jiahui Han[2], Jiang Li[2], Ruyu Gao[2], Zhengfu Zhou[2], Wei Zhang[2], Ming Chen[2], Min Lin[2] , Jinwang[2*], Kaijing Zuo[1*],

1 Department of Plant Science, School of Agriculture and Biology, Shanghai Jiao Tong University, Shanghai 200240, China.
chenyun0402ye@163.com (Y.C); sunwenjie_007@163.com (W.S); kjzuo@sjtu.edu.cn (K.Z)

2 Biotechnology Research Institute, Chinese Academy of Agricultural Sciences, Beijing, China;
xue_dong_kevin@126.com (D.X.); 13121257599@163.com (J.H.); 1329090356@qq.com (J.L.); rainie0327@mail.dlut.edu.cn (R.G); zhouzhengfu@caas.cn (Z.Z.); zhangwei01@caas.cn (W.Z.); chenming01@caas.cn (M.C.); linmin57@vip.163.com (M.L.); wangjin@caas.cn (J.W.)

* Correspondence: wangjin@caas.cn; kjzuo@sjtu.edu.cn

Supplementary Table 1. Small RNA sequencing and classification in *D. radiodurans* R1.

Sequencing data	Control (CDR)	H_2O_2 treatment (TDR)
Number of raw reads	38,097,349	44,092,613
Number of clean reads	37,603,677	43,287,070
Percentage of clean reads	98.70%	98.17%
Size of clean reads	5.64 Gbp	6.49 Gbp

Supplementary Table 2. Information of 24 sRNAs involved in oxidative stress response..

IGR_ID	Start	End	upgene	downgene	strand	foldchange	p-value	q-value
IGR_3053	358768	358969	DR_A0243	DR_A0244	+	0.718681	1.57E-08	4.14E-09
IGR_2205	678996	678583	DR_2032	DR_2031	_	-1.03092	2.92E-10	8.35E-11
IGR_1662	1218295	1217830	DR_1490	DR_1489	_	0.069874	0.733049	0.09144
IGR_1449	1437594	1437664	DR_1275	DR_1276	+	-2.71252	0	0
IGR_1916	953162	952832	DR_1743	DR_1742	_	-0.14034	0.079399	0.011909
IGR_1612	1279547	1279884	DR_1439	DR_1440	+	0.92522	5.79E-103	6.59E-103
IGR_2590	305214	305487	DR_2417m	DR_2418	+	-1.99071	0	0
IGR_884	1994107	1994359	DR_0713	DR_0714	+	0.112089	0.495287	0.064129
IGR_2572	323499	323822	DR_2399	DR_2400	+	-0.96447	2.76E-17	1.02E-17
IGR_2408	494640	494504	DR_2235	DR_2234	_	-1.50432	1.77E-19	6.99E-20
IGR_771	2113157	2113436	DR_0600	DR_0601	+	-1.04859	5.86E-51	3.90E-51
IGR_2060	816490	816280	DR_1887	DR_1886	_	0.082214	0.421874	0.055479
IGR_1174	1704381	1704423	DR_1002	DR_1003	+	-0.22	0.297456	0.040242
IGR_2479	419902	420112	DR_2306	DR_2307	+	-0.78763	1.76E-05	3.80E-06

IGR_2150	725671	725829	DR_1976	DR_1977	+	-0.73333	5.43E-24	2.38E-24
IGR_2690	207317	207620	DR_2516	DR_2517	+	-0.30675	0.230451	0.031796
IGR_2389	510360	510248	DR_2217	DR_2216	_	-0.04801	0.505675	0.065342
IGR_76	160671	160543	DR_B0087	DR_B0086	_	/	/	/
IGR_585	2309594	2309548	DR_0415	DR_0414	_	-1.20345	7.47E-26	3.40E-26
IGR_2012	856316	856568	DR_1838	DR_1839	+	0.717014	1.26E-44	7.77E-45
IGR_373	2519079	2519185	DR_0202	DR_0203	+	/	/	/
IGR_826	2058350	2058826	DR_0655	DR_0656	+	0.787837	0.000166	3.32E-05
IGR_1951	916929	916729	DR_1778	DR_1777	_	2.194254	1.30E-305	3.92E-305
IGR_388	2506155	2505865	DR_0218	DR_0217	_	0.232319	6.77E-24	2.96E-24

Supplementary Table3. KEGG pathway analysis of oxidative response sRNAs in *D. radiodurans R1*.

Pathway	Pathway ID.	sRNA/Product of target gene pair	P-value	Q-value
Peroxisome	K04146	OsiA/DR_1998; OsiA/DR_A0202<br>IGR_1662/DR_A0259;IGR_2590/DR_A0309<br>IGR_1662/DR_1168; IGR_1912/DR_1279<br>IGR_2205/DR_1350; IGR_1174/DR_1540<br>IGR_2389/DR_1546; IGR_1916/DR_1692	0.6787235	9.848279e-01
Mismatch repair	KO03430	IGR_1612/DR_0001; IGR_76/DR_0099<br>IGR_1662/DR_0100; IGR_884/DR_0186<br>IGR_585/DR_0315; OsiA/DR_0316<br>IGR_2479/DR_0507; IGR_2572/DR_0607<br>IGR_2150/DR_0822; IGR_1612/DR_0856<br>IGR_1662/DR_1039; IGR_2012/DR_1126<br>IGR_1916/DR_1244; IGR_1449/DR_1696<br>IGR_1449/DR_1775; IGR_2590/DR_1778<br>IGR_2408/DR_1943; IGR_2572/DR_1976<br>IGR_2205/DR_2069; IGR_2205/DR_2410<br>IGR_2205/DR_2586	0.4417978	9.848279e-01
Glutathione metabolism	Ko00480	IGR_1916/DR_0148; IGR_2690/DR_0651<br>IGR_771/DR_0692; IGR_771/DR_0717<br>IGR_2150/DR_1524; IGR_1174/DR_1540<br>IGR_2690/DR_1595; IGR_2205/DR_1596<br>IGR_884/DR_1758; IGR_1449/DR_B0087	0.6787235	9.848279e-01
Carotenoid biosynthesis	Ko00906	IGR_771/DR_0801; IGR_2205/DR_0861<br>IGR_1662/DR_0862	0.8904877	9.848279e-01
Two-component system	Ko02020	IGR_1662/DR_0023; IGR_1662/DR_0024<br>IGR_1662/DR_0926; IGR_2205/DR_0927<br>IGR_373/DR_0950; IGR_826/DR_0951<br>IGR_1662/DR_0952; IGR_2479/DR_0954	0.08660762	9.848279e-01

		IGR_2205/DR_A0009;IGR_1951/DR_A0010<br>IGR_388/DR_A0350; IGR_2572/DR_A0351<br>IGR_2408/DR_A0352;IGR_2205 /DR_A0353<br>OsiA/DR_A0354; IGR_2408/DR_A0355<br>IGR_884/DR_B0083; IGR_2060/DR_B0086<br>IGR_1449/DR_B0087; IGR_1449/DR_B0088		
Vitamin B6 metabolism	Ko00750	IGR_1449/DR_0341; IGR_2205/DR_0495<br>IGR_2060/DR_0596; IGR_1612/DR_1274<br>IGR_1449/DR_1366; IGR_2060/DR_1367<br>IGR_2590/DR_A0184;IGR_2408/DR_A0360	0.7335775	9.848279e-01

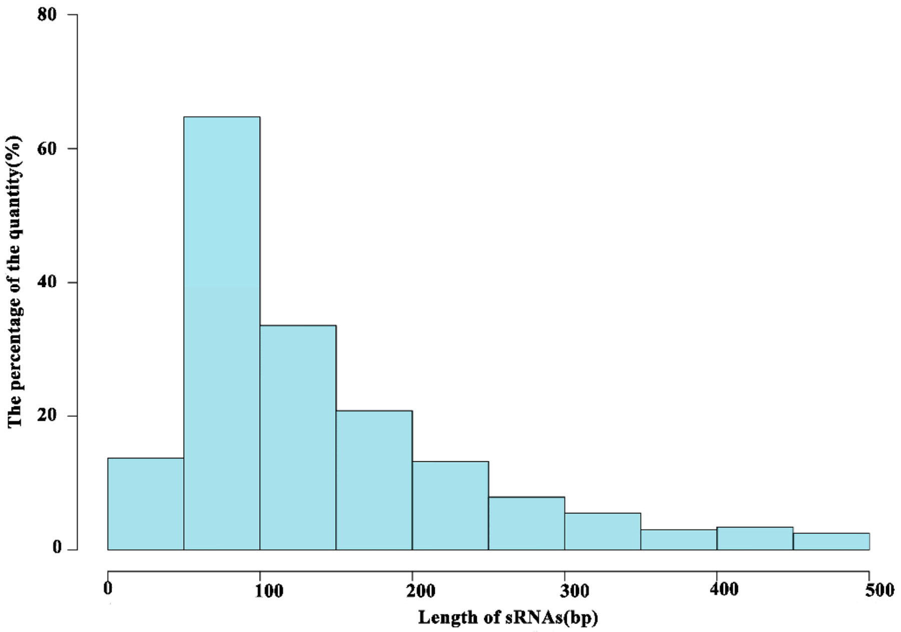

Supplementary Figure 1. The sRNA length distribution in *D. radiodurans R1*. The sRNA length (nt) is shown on the x-axis and the percentage of sRNA quantity is represented on the y-axis.

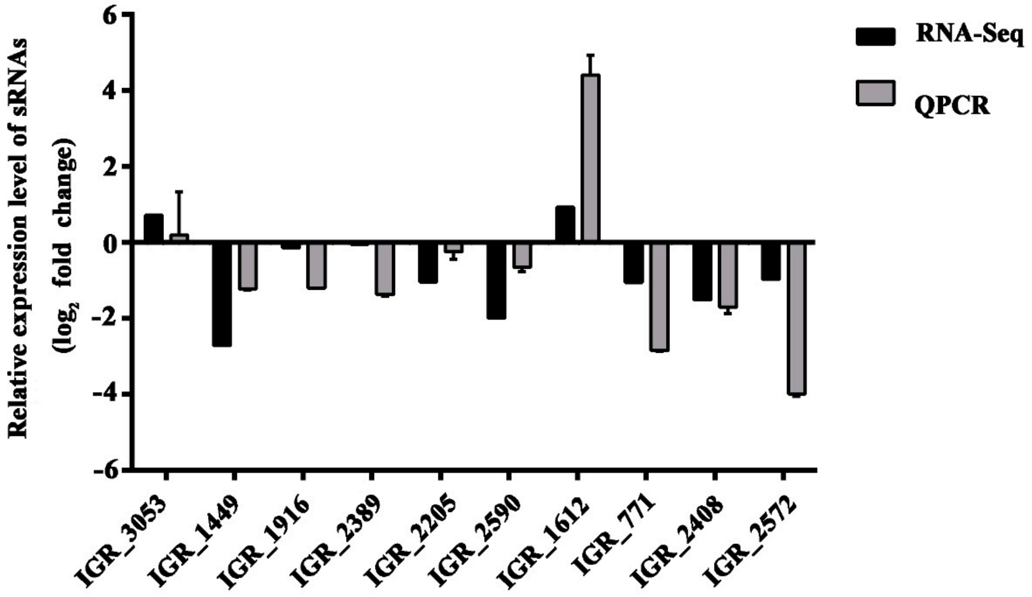

Supplementary Figure 2. qRT-PCR validation of 10 sRNAs identified by RNA-seq. Vertical values represent fold change (log_2) of small noncoding RNAs (sRNAs) at the transcriptional level after H_2O_2 treatment.

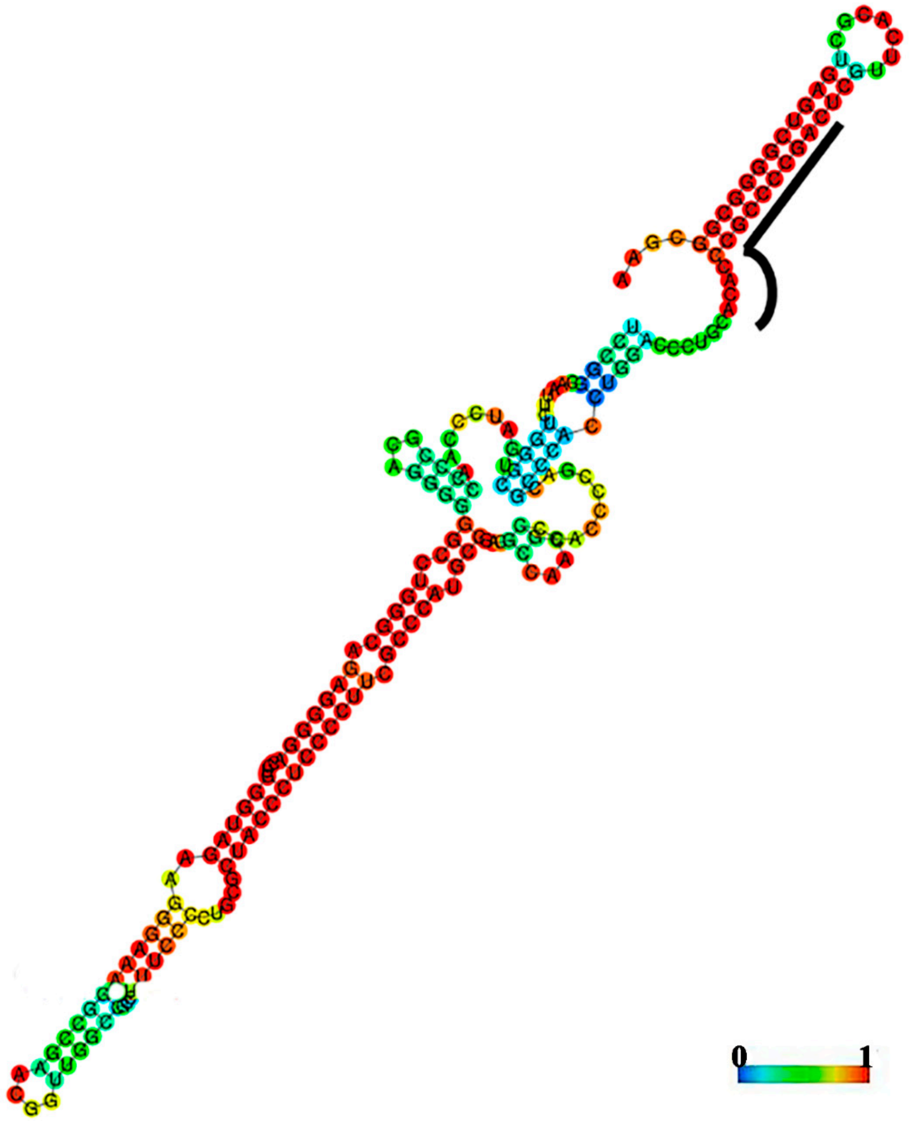

Supplementary Figure 3. The deduced second structure of OsiA. The black line in the figure represents the common areas on OsiA where each target gene is combined. The color legends represent base pair probabilities.
